# Single-cell extracellular vesicle-program scoring maps immunometabolic rewiring and immune crosstalk of mesenchymal stromal cells in intervertebral disc degeneration, prioritizing AP2S1 and CSTB

**DOI:** 10.3389/fimmu.2026.1820174

**Published:** 2026-05-22

**Authors:** Fengyu Ma, Kelv Shen, Ziqiang Wu, Hanshi Yang, Mingzhe Yu, Zhendong Huang, Fengxuan Han, Zhengfeng Lu

**Affiliations:** 1 Department of Orthopedics, The Second Affiliated Hospital of Soochow University, Suzhou, Jiangsu, China; 2 Department of Spinal Surgery, People’s Hospital of Rizhao, Rizhao, Shandong, China; 3 Medical 3D Printing Center, Orthopedic Institute, Department of Orthopedic Surgery, The First Affiliated Hospital, School of Basic Medical Sciences, Interdisciplinary Innovation Center for Nanomedicine, MOE Key Laboratory of Geriatric Diseases and Immunology, Suzhou Medical College, Soochow University, Suzhou, Jiangsu, China; 4 Children’s Hospital of Soochow University, Suzhou, Jiangsu, China

**Keywords:** immunometabolic reprogramming, intervertebral disc degeneration, machine learning, mesenchymal stromal cells, MSC EV-program scoring, single-cell RNA sequencing

## Abstract

**Background:**

Intervertebral disc degeneration (IDD) is increasingly viewed as an immune-perturbed and metabolically stressed niche rather than a purely mechanical or aging-related disorder. Mesenchymal stromal cells (MSCs) and their extracellular vesicles (EVs) may shape local immune signaling, yet MSC EV–associated transcriptional programs and their immune-network context remain poorly defined at single-cell resolution.

**Methods:**

We analyzed single-cell RNA sequencing (scRNA-seq) data from GSE230809 (8 IDD and 3 control nucleus pulposus samples) with standard preprocessing and batch correction. We derived a transcriptome-inferred MSC EV–associated program score by integrating complementary gene-set scoring strategies, stratified MSCs into EV-score–high versus –low states, and prioritized candidate regulators using a consensus LASSO/SVM-RFE workflow. CellChat was used to infer ligand–receptor communication, while pathway-level immunometabolic remodeling was assessed with GSVA/GSEA and immune-signature correlation analyses. Selected genes were examined by RT–qPCR in an *in vitro* inflammatory NP-cell model, and docking was performed to explore tractable compound–target hypotheses.

**Results:**

We resolved seven major cell populations and observed a marked shift toward higher EV-program scores in MSCs from IDD tissues. EV-score–high MSCs showed increased incoming signaling and interaction potential in CellChat networks, consistent with an “immune cue–responsive” state. Machine-learning prioritization converged on five hub genes (AP2S1, CSTB, GSTP1, RPL28, and TSG101). The hub-gene program aligned with immune–metabolic axes including IL6/JAK–STAT3 and interferon-related signaling, together with oxidative phosphorylation and ROS stress signatures, and was systematically associated with immune mediators (notably chemokines and checkpoint-related genes). AP2S1 and CSTB displayed dynamic expression along MSC pseudotime trajectories and were supported by RT–qPCR under inflammatory stimulation. A hub-gene model distinguished IDD from control samples within the discovery cohort (AUC = 0.836). Docking suggested a plausible interaction between CSTB and ripasudil (−5.98 kcal/mol).

**Conclusions:**

This study maps an MSC EV–associated program at single-cell resolution and places it within an immune–communication and immunometabolic framework in IDD. AP2S1 and CSTB are candidate nodes linking EV-program states to immune signaling and metabolic stress, providing candidate biomarkers and tractable intervention hypotheses.

## 1 Introduction

Low back pain (LBP) is among the most prevalent musculoskeletal disorders worldwide. In 2020, an estimated 619 million individuals were affected; this number is projected to reach 843 million by 2050 (1). Intervertebral disc degeneration (IDD) is widely regarded as a principal pathological substrate of LBP (1, 2). Mild disc degeneration has been reported in approximately 20% of adolescents, and up to 80% of people experience at least one episode of LBP over their lifetime, with annual treatment and rehabilitation costs exceeding US$100 billion (3, 4). The intervertebral disc (IVD) consists of a central nucleus pulposus (NP), surrounding annulus fibrosus (AF), and superior and inferior cartilaginous endplates (CEPs). These structures support spinal load distribution and flexibility. IDD is characterized by AF disruption, NP dehydration, and imbalance of extracellular matrix (ECM) composition, accompanied by increased expression of ECM-degrading enzymes, including matrix metalloproteinases (MMPs) and a disintegrin and metalloproteinase with thrombospondin motifs (ADAMTS) family members (5, 6). As degeneration progresses, NP and AF cells exhibit reduced synthesis of type II collagen and proteoglycans, leading to declining tissue hydration and, ultimately, loss of disc height and structural collapse (5, 7).

Previous studies have shown that IDD is not merely a consequence of mechanical loading and aging; immune and inflammatory processes contribute to its initiation and progression (6, 8). Under physiological conditions, the intervertebral disc is an enclosed, avascular tissue that functions as an immune-privileged niche (2, 7). During degeneration, fissures in the cartilage endplate and AF permit immune-cell infiltration—including macrophages, T cells, and neutrophils—into the NP compartment (7, 9). These infiltrating cells secrete pro-inflammatory mediators such as IL-1β, TNF-α, and IL-6, which accelerate ECM breakdown and cellular injury (6, 8). Macrophages with distinct M1/M2 polarization states can release matrix-degrading enzymes (e.g., MMP-9 and MMP-13) as well as inflammatory cytokines (TNF-α and IL-1β), directly contributing to disc catabolism and degeneration (7, 10). At the intracellular level, key signaling cascades implicated in these processes include NF-κB, MAPK, JAK/STAT3, and the NLRP3 inflammasome pathway (6, 8). NP cells may undergo apoptosis or inflammatory pyroptosis, further amplifying degenerative progression (3, 9). The degenerative disc microenvironment is characterized by immune-driven dysregulation of matrix metabolism, and “immunometabolic reprogramming” has emerged as a rapidly expanding conceptual framework in IDD research (2, 6).

Current treatments for IDD, including analgesics, physical therapy, and surgery, mainly relieve symptoms and have limited capacity to restore native disc structure or function. Given their multilineage differentiation capacity and potent immunomodulatory functions, mesenchymal stem/stromal cells (MSCs) have become a focal point of regenerative strategies for IDD (4, 11). Accumulating evidence indicates that much of the therapeutic benefit of MSCs is mediated not by engraftment, but by paracrine signaling—most notably through mesenchymal stromal/stem cell–derived extracellular vesicles (MSC-EVs) (11, 12). Compared with currently available therapies and direct live-cell transplantation, MSC-EV–based approaches may offer advantages, including lower immunogenicity, reduced risks associated with live-cell administration, and greater feasibility for storage, standardization, and delivery. MSC-derived EVs package bioactive cargoes, including proteins, mRNAs, and microRNAs, and thereby reprogram the functional state of recipient cells (11, 12). Across animal models and *in vitro* systems, MSC-EVs have been shown to enhance NP cell proliferation, promote ECM anabolism, and attenuate inflammation and apoptosis (12, 13). For example, Zhao and colleagues reported that MSC-EVs released by MSCs under hypoxic and inflammatory priming delivered miR-221-3p to suppress the DDIT4–NF-κB axis, mitigating senescence of NP cells (14). Peng and colleagues showed that EVs enriched in the antioxidant enzyme SOD1 dampened NLRP3 inflammasome activation in NP cells, protecting them from pyroptotic injury (13). These findings support a role for MSC-EVs in shaping disc inflammation, oxidative stress, and cell fate, yet the underlying mechanisms—and the key molecular determinants—remain incompletely defined (13, 14).

As single-cell sequencing has matured, an increasing number of studies have begun to resolve cellular heterogeneity and intercellular communication in IDD tissues. Ling and colleagues profiled human NP across degenerative stages by single-cell RNA sequencing, identifying multiple NP cell states—such as metabolic homeostatic, inflammatory-responsive, and fibrocartilaginous programs—alongside diverse immune populations (9, 15). They reported prominent macrophage–NP stem/progenitor cell crosstalk in advanced degeneration, mediated through MIF-linked NF-κB signaling, suggesting that immune-derived cues can actively sculpt the NP microenvironment (9, 15). Complementing these findings, Zhang and colleagues showed that a chondrocyte subset with a fibrotic bias engages macrophages and vascular-associated cells in feed-forward inflammatory amplification (15). These studies support a shared view: cell–cell interactions—particularly communication between immune cells and disc-resident cells—are integral to the pathobiology of IDD. In this context, CellChat, developed by Jin and colleagues, enables quantitative inference of ligand–receptor–based communication networks from single-cell datasets (16), providing a methodological framework for interrogating signaling exchange across cell types.

Despite substantial progress in charting the cellular composition of disc tissues and their communication networks, single-cell interrogation of MSC EV-associated programs remains limited. In particular, current single-cell frameworks rarely provide a principled way to quantify “MSC EV–associated transcriptional activity” at the level of individual cells and attempts to couple such a metric with machine-learning–driven prioritization of core regulators are largely absent. Moreover, how MSC-EV programs reshape the disc microenvironment through immunometabolic pathways has yet to be clarified. Here, we introduce a single-cell MSC EV-program score to functionally stratify MSCs on a cell-by-cell basis. The EV-program score used here quantifies an EV-associated transcriptional program rather than directly measuring EV secretion, composition, or therapeutic efficacy. Therefore, our study is designed to prioritize regulators and hypotheses that can be experimentally tested in MSC-EV functional systems, rather than to claim definitive repair by exogenous MSC-EVs. By integrating this scoring scheme with complementary machine-learning feature selection (least absolute shrinkage and selection operator (LASSO) and support vector machine–recursive feature elimination (SVM-RFE)), we identify MSC-EVs-linked key regulators, including AP2S1 and CSTB. We further delineate intercellular signaling with CellChat and, through pathway enrichment analyses, connect the resulting gene program to major immunometabolic axes, including IL6/JAK–STAT3, PI3K–Akt, p53 signaling, oxidative phosphorylation, and reactive oxygen species (ROS)-related pathways, implicating these regulators in immune-metabolic control of disc homeostasis. Consistently, ROC analyses and predictive modeling showed within-cohort discriminatory performance for the signature (AUC = 0.836). Molecular docking suggested that the small molecule ripasudil forms a stable interaction with CSTB, highlighting a potential avenue for therapeutic development.

We coupled a single-cell MSC EV-program scoring framework with machine-learning prioritization to examine how MSC-EV programs relate to immunometabolic regulation in IDD. AP2S1 and CSTB provide candidate entry points for testing how MSC EV-linked programs interact with immune and metabolic stress in IDD. Beyond refining the immunological view of the degenerative disc microenvironment, our findings provide a mechanistic rationale for optimizing MSC-based therapies.

## 2 Methods

### 2.1 Data acquisition

Single-cell RNA-seq data were obtained from GEO (GSE230809), including NP tissues from 11 individuals (IDD, n = 8; controls, n = 3). For sample-level external validation, we retrieved GSE70362 (platform GPL17810), which included 24 NP specimens (IDD, n = 16; controls, n = 8). All analyses used publicly available, de-identified data; no additional ethics approval was required for secondary analysis. For independent external validation of the prioritized hub genes, we additionally analyzed GSE186542, a human NP bulk transcriptomic cohort including early degeneration (Pfirrmann grade I–III, n = 3) and late degeneration (grade IV–V, n = 3). This dataset was used exclusively to assess out-of-cohort diagnostic performance of the hub genes by ROC analysis.

### 2.2 Single-cell transcriptomic analysis

All scRNA-seq analyses were performed in Seurat. Cells expressing at least 200 genes were retained, and genes detected in fewer than 3 cells were removed. Cells with mitochondrial transcript proportions above the prespecified threshold were excluded. After LogNormalize and scaling, 2,000 highly variable genes were selected for PCA. Batch effects across individuals were corrected with Harmony using Harmony-corrected principal components with default parameters. Clustering was performed on the Harmony-corrected space, and Uniform Manifold Approximation and Projection (UMAP) was used for visualization. Differential markers across clusters were identified with FindAllMarkers (assay = “RNA”, min.pct = 0.25, logfc.threshold = 0.585). Cell types were assigned based on canonical markers and cluster-level differential expression, resulting in seven major populations (MSCs, NP cells, fibrochondrocytes, fibroblasts, regulatory chondrocytes, stem cells, and progenitor cells).

### 2.3 EV-associated program scoring and MSC stratification

An EV-associated gene set (121 genes; curated from ExoBCD) was used to quantify transcriptome-inferred EV-program activity at single-cell resolution. Five complementary scoring methods were computed: AUCell, UCell, singscore, ssGSEA, and AddModuleScore. For each method, scores were min–max normalized to [0, 1], and an integrated EV-program score (Scoring) was defined as the sum of the five normalized scores per cell. MSCs were stratified into EV-score–high and EV-score–low groups using the median MSC Scoring value.

### 2.4 Cell–cell communication inference

Cell–cell communication was inferred using CellChat with the human ligand–receptor database. The CellChatDB.human database was used, restricted to the “Secreted Signaling” category. Normalized expression and Seurat-derived cell labels were used as input. Communication probability and network centrality metrics were computed under default settings unless stated otherwise. To compare MSC subgroups, MSCs were split into EV-score–high and EV-score–low identities and included as distinct nodes in the same network; incoming/outgoing signaling strengths and interaction counts were then summarized for each node.

### 2.5 Feature selection

EV-score-linked genes from the high-vs-low MSC comparison were used as candidate features. Feature selection was performed by two independent methods: (i) LASSO with 10-fold cross-validation to determine lambda, and (ii) SVM-RFE with cross-validation-based error minimization. The intersection of features selected by both methods was retained as the consensus hub-gene set.

### 2.6 Reverse transcription–quantitative polymerase chain reaction validation in an IDD-like NP cell model

Human primary NP cells (catalog no. HUM-iCell-s012; Cellverse Co., Ltd.; passage 1; cryopreserved) were stimulated with LPS (1 µg/mL, 48 h) to establish an IDD-like inflammatory condition. Total RNA was extracted, reverse-transcribed, and quantified by RT–qPCR (SYBR Green). Relative expression was calculated using the 2^−ΔΔCt^ method with GAPDH as the reference gene.

### 2.7 Immune infiltration estimation (bulk dataset)

Immune infiltration was estimated in GSE70362 using single-sample Gene Set Enrichment Analysis (ssGSEA). We used a curated immune signature set comprising 29 immune categories and 547 marker genes. ssGSEA scores were compared between controls and IDD samples using the Wilcoxon rank-sum test with FDR correction.

### 2.8 Pathway-level analyses (gene set variation analysis/gene set enrichment analysis)

GSVA was performed using MSigDB Hallmark gene sets to obtain per-sample pathway activity scores. For each key gene, samples were split into high vs low groups by the median expression, and differential GSVA scores were tested using limma with FDR correction. GSEA was performed on ranked gene lists using MSigDB collections; pathways with adjusted *p* < 0.050 were considered significant.

### 2.9 Predictive model, nomogram, and external validation of hub genes

A logistic regression model was fitted using the selected hub genes. A nomogram was constructed from the fitted coefficients. Model performance was evaluated by ROC/AUC analysis. AUC was computed within the same dataset and should be interpreted as within-cohort performance.

For external validation, ROC analysis was performed in GSE186542. Samples were classified as early degeneration (Pfirrmann I–III) or late degeneration (Pfirrmann IV–V). For each hub gene, expression values were used as predictors and degeneration status as the response. ROC curves and area under the curve (AUC) values with 95% confidence intervals were calculated using the pROC package in R. Given the limited sample size of the external cohort, these analyses were interpreted as supportive validation rather than definitive performance estimates.

### 2.10 Molecular docking

Protein structures were retrieved from AlphaFold. Candidate drug–target links were queried in DGIdb, and ligand structures were obtained from PubChem. Docking was performed using AutoDock4. For each ligand–protein pair, 50 independent runs were performed, and the lowest-energy pose was retained for visualization in PyMOL.

### 2.11 Statistics

Statistical analyses were performed in R (v4.3.2). Two-group comparisons used Wilcoxon rank-sum tests unless otherwise stated. Correlations were computed using Spearman’s rank correlation. *p* values were adjusted by the Benjamini–Hochberg method when multiple testing was involved. A two-sided adjusted *p* < 0.050 was considered significant.

## 3 Results

### 3.1 Single-cell atlas of NP tissues in IDD

**Figure 1:**
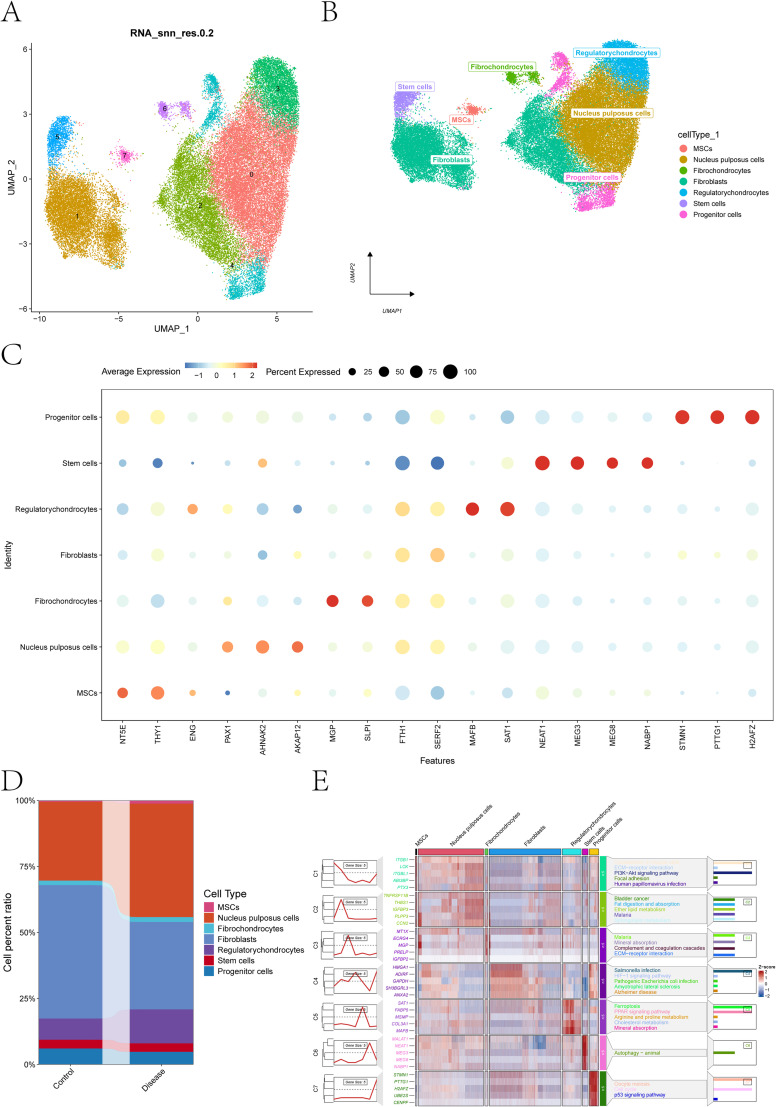
Cell-type annotation and functional signatures across single-cell clusters. **(A)** UMAP projection based on harmony-corrected principal components, resolving seven transcriptionally distinct populations. **(B)** cell-type annotation of the seven clusters, assigned to major disc-resident populations including MSCs, NP cells, fibrochondrocytes, fibroblasts, regulatory chondrocytes, stem cells, and progenitor cells. **(C)** dot plot summarizing canonical marker expression across the annotated cell types; dot size denotes the fraction of cells expressing each marker, and color indicates scaled average expression. **(D)** relative proportions of each annotated cell population across samples/groups. **(E)** clusterGVis-based functional enrichment heatmap and annotation for average expression profiles across cell subpopulations. Each row block represents a gene module; the central heatmap displays module expression patterns across cell types (Z-score–normalized), and the right panel shows corresponding KEGG pathway enrichments. Colors indicate pathway categories, and bar length reflects enrichment significance.

We analyzed GSE230809 (IDD, n = 8; controls, n = 3). After quality control (≥200 genes per cell; mitochondrial fraction cutoff as specified in Methods), cells were retained for downstream analyses (**Supplementary Figure 1**). Harmony integration reduced inter-individual batch separation in the PCA space, enabling clustering on a shared manifold. UMAP resolved seven major NP-resident populations (**Figures 1A,B**). Canonical markers supported the assigned identities (**Figure 1C**), and group-wise cell composition is shown in **Figure 1D**. Pathway-level module visualization (ClusterGVis) highlighted enrichment of PI3K–Akt signaling and cytoskeletal programs in MSC-associated modules (**Figure 1E**).

### 3.2 EV-associated program scoring identifies an MSC-shift in IDD

**Figure 2:**
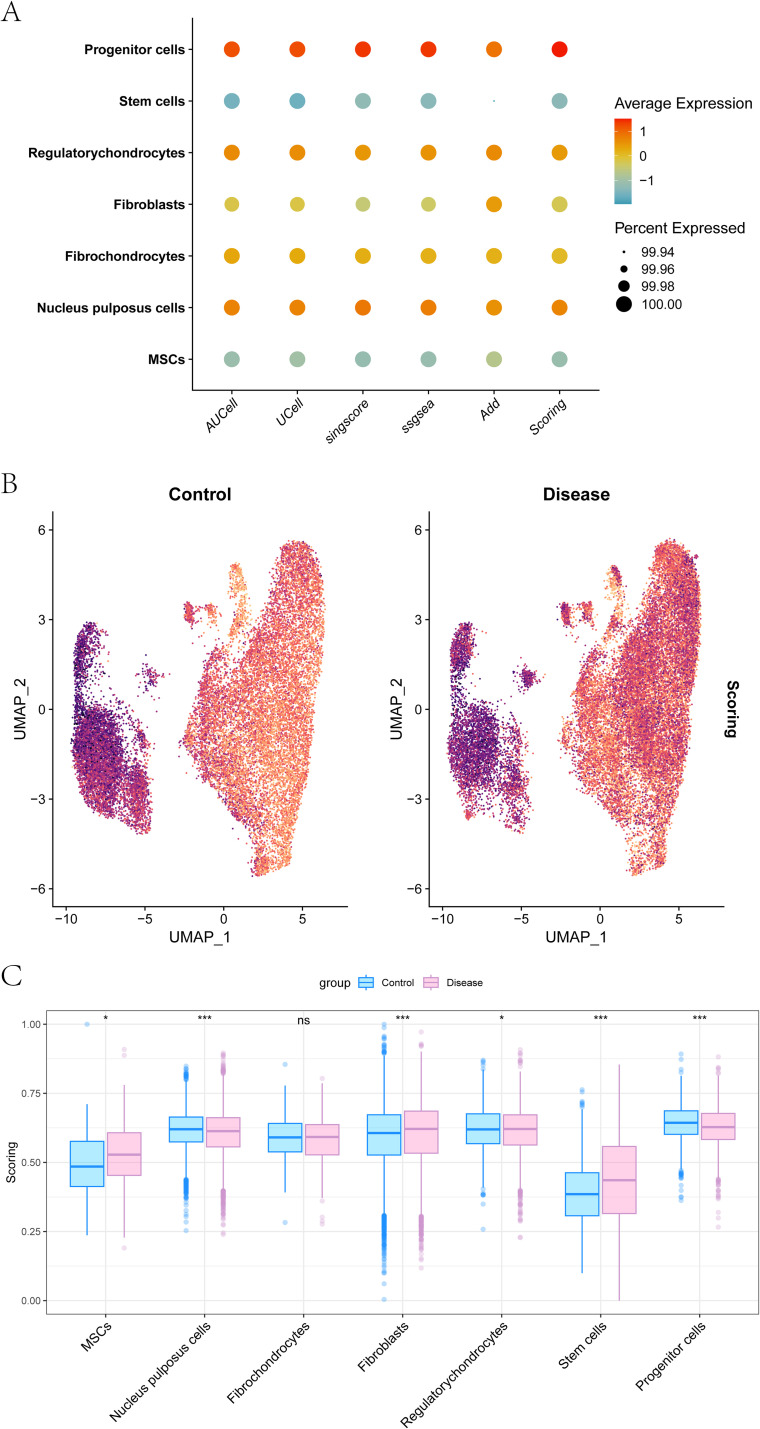
Single-cell characterization of MSC EV-program activity. **(A)** dot plot of MSC EV-related gene-set scores across seven cell subpopulations computed using five complementary methods (AUCell, UCell, singscore, ssGSEA, and AddModuleScore) together with the integrated scoring metric. Dot color indicates the Z-score–scaled average gene-set activity, and dot size denotes the fraction of cells with detectable activity (percent expressed). **(B)** UMAP visualization of EV-associated program stratified by disease status (control vs disease) using the integrated MSC EV-program score. Color intensity reflects per-cell scoring magnitude (scores are transcriptome-inferred). **(C)** comparison of scoring distributions across cell subpopulations between control (blue) and disease (pink). Box plots summarize score distributions. Statistical significance is denoted as: *p < 0.05; **p < 0.01; ***p < 0.001. “ns” indicates not significant.

We curated 121 EV-associated genes from ExoBCD and quantified transcriptome-inferred program activity using ssGSEA, AUCell, UCell, singscore, and AddModuleScore, followed by an integrated EV-score (**Figure 2A**). Among cell types, MSCs showed the clearest disease-associated shift: EV-scores were higher in IDD than in controls (**Figures 2B,C**). We then split MSCs at the median EV-score and performed within-MSC differential expression. Using |avg_log2FC| > 0.500 and FDR < 0.050, we obtained 16 EV-score–linked genes. The EV-score reflects coordinated expression of EV-associated genes and does not represent a direct readout of EV secretion or functional potency.

### 3.3 EV-score–high MSCs show increased incoming signaling in CellChat networks

**Figure 3:**
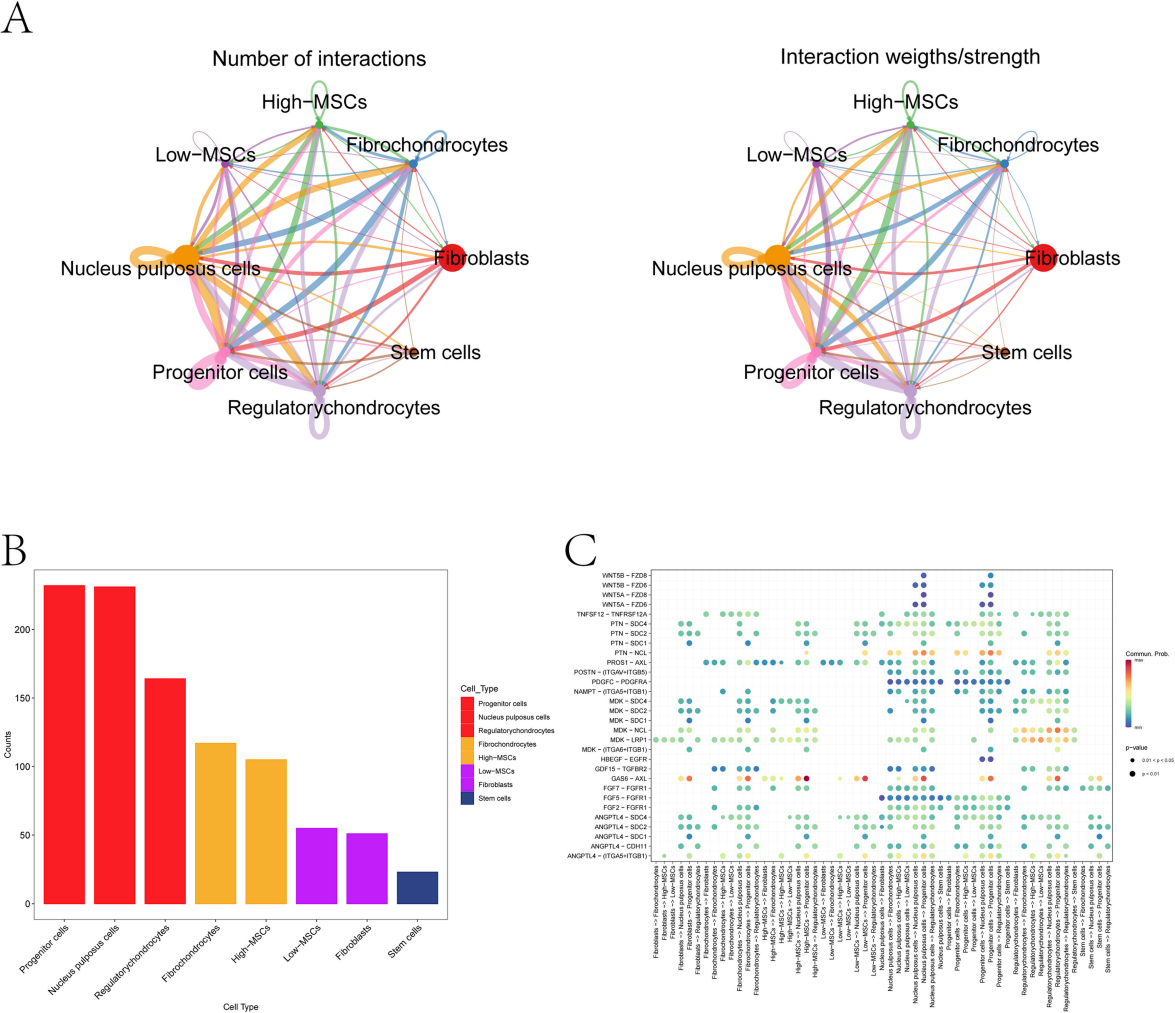
Ligand–receptor communication inferred by CellChat. **(A)** global cell–cell communication network across annotated populations. Edge width represents the inferred communication probability/interaction strength between cell types. **(B)** incoming/outgoing strength for EV-score–high versus EV-score–low MSCs (ordered from highest to lowest, left to right). **(C)** bubble plot of inferred ligand–receptor interactions between cell populations. Bubble size reflects interaction strength (or communication probability), and color encodes relative interaction intensity as defined in the analysis pipeline.

CellChat inferred ligand–receptor communication among NP-resident populations (**Figure 3A**). When MSCs were split by EV-score, EV-score–high MSCs displayed higher incoming signaling strength and more incoming interactions than EV-score–low MSCs (**Figure 3B**). The bubble plot showed dominant ligand–receptor pairs received by EV-score–high MSCs (**Figure 3C**).

### 3.4 LASSO and SVM-RFE converge on five hub genes

**Figure 4:**
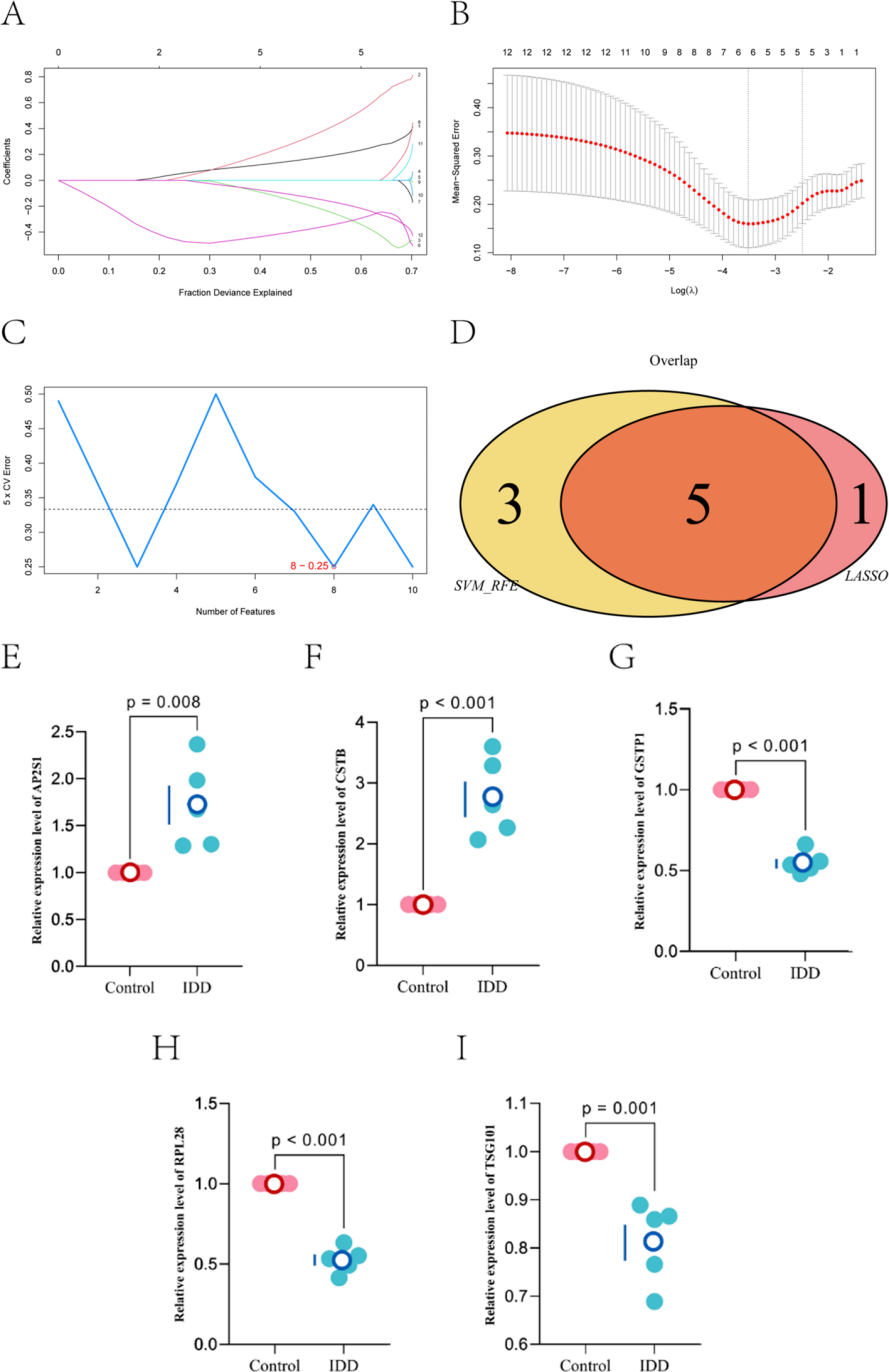
Construction of LASSO and SVM-based predictive features and experimental validation. **(A)** LASSO coefficient profiles and the selected gene set at the optimal (minimum) λ. **(B)** ten-fold cross-validation for tuning-parameter selection in the LASSO model to identify the minimum λ. **(C)** feature selection by SVM. The red-highlighted point denotes the feature number corresponding to the lowest classification error. **(D)** Venn diagram showing the intersection of candidate features selected by LASSO and SVM, yielding the consensus gene set. **(E–I)** expression of AP2S1, CSTB, GSTP1, RPL28, and TSG101 in human NP cells under basal (normal) conditions and under degenerative (LPS-stimulated) conditions.

We used the 16 EV-score–linked genes as the candidate pool. LASSO logistic regression selected six features at the optimal λ (**Figures 4A,B**), whereas SVM-RFE identified eight features with the lowest classification error (**Figure 4C**). The overlap yielded five hub genes: AP2S1, CSTB, GSTP1, RPL28, and TSG101 (**Figure 4D**).

### 3.5 RT–qPCR validation in an inflammatory NP-cell model

Human NP cells were stimulated with LPS and compared with basal controls. RT–qPCR showed higher AP2S1 and CSTB expression under LPS stimulation, whereas GSTP1, RPL28, and TSG101 were lower (**Figures 4E–I**).

### 3.6 Immune infiltration signatures associate with AP2S1/CSTB in bulk NP transcriptomes

**Figure 5:**
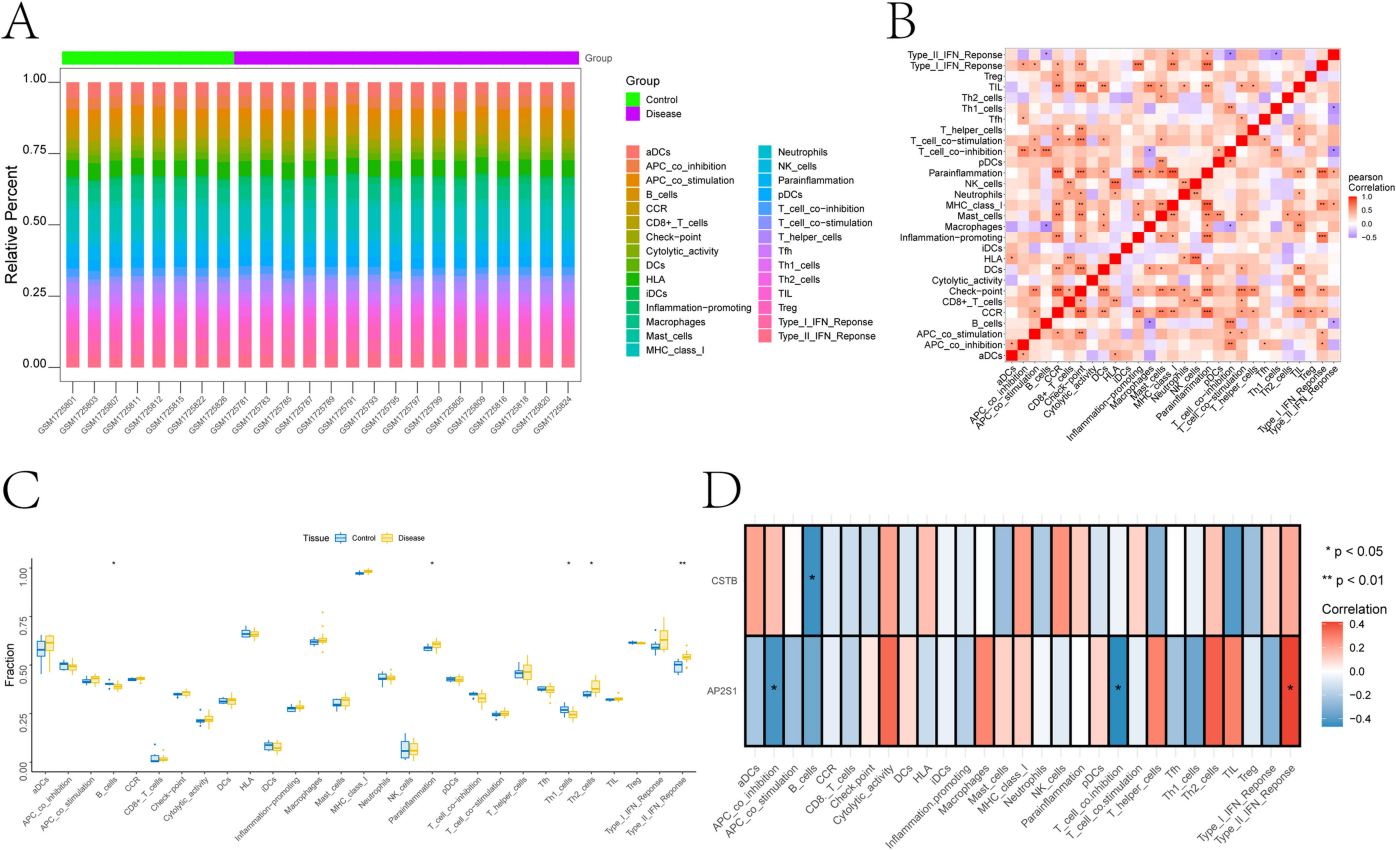
Immune infiltration landscape and associations with key genes. **(A)** relative proportions of inferred immune-cell subsets across samples. **(B)** pairwise correlations among immune-cell signatures; blue indicates negative correlation and red indicates positive correlation. **(C)** differential abundance of immune-cell phenotypes between control and disease samples. **(D)** correlations between key-gene expression and immune-cell infiltration signatures.

In GSE70362, ssGSEA showed higher immune-signature scores in IDD samples than in controls (**Figures 5A–C**). AP2S1 showed a negative correlation with T cell co-inhibition and positive correlation with type II interferon (IFN) response (**Figure 5D**). CSTB was inversely correlated with B cell signature (**Figure 5D**).

**Figure 6:**
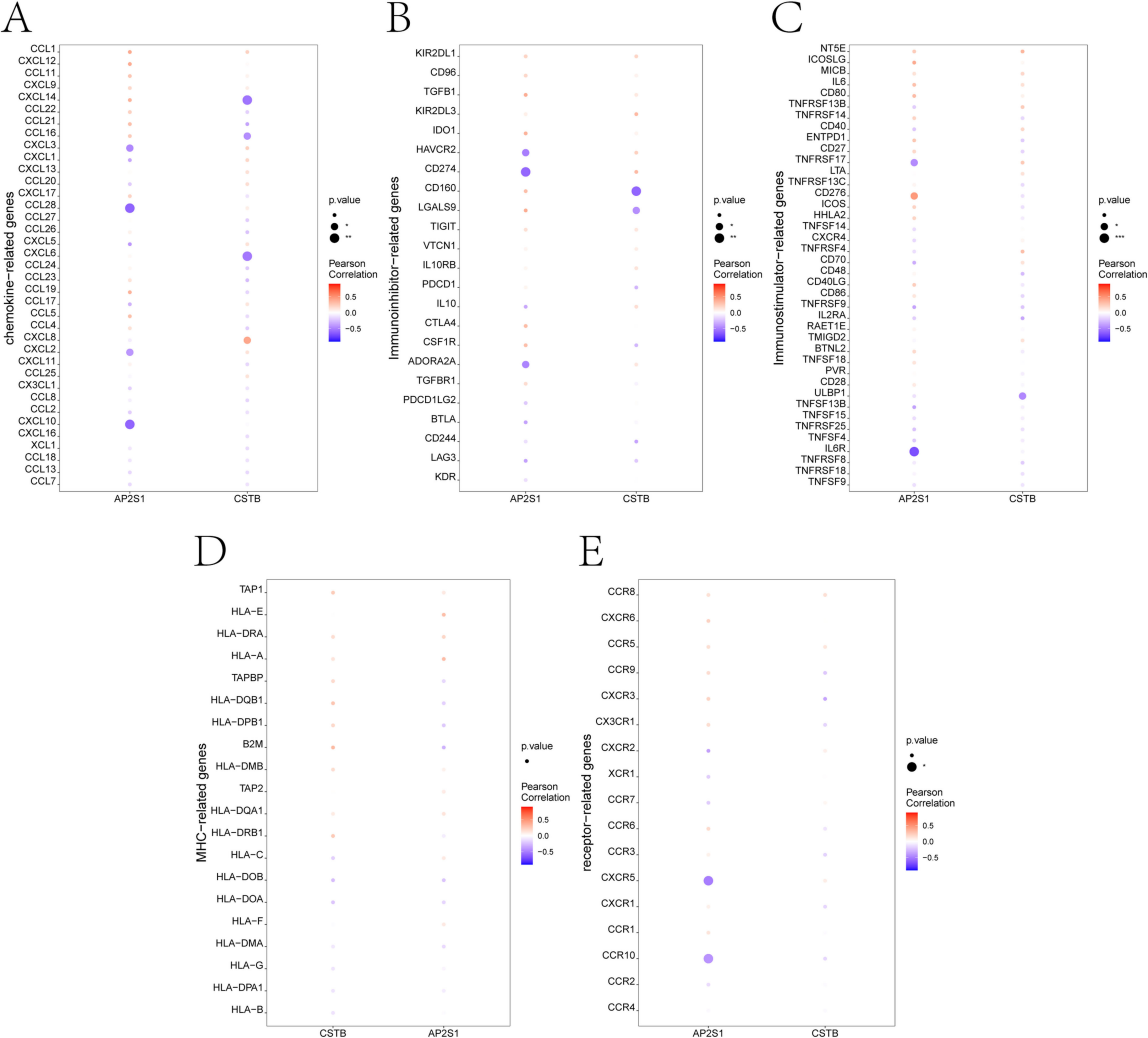
Relationships between key genes and immune mediators. **(A–E)** correlation analyses between key-gene expression and immune mediators, including chemokines, immunoinhibitors, immunostimulators, major histocompatibility complex (MHC)-related molecules, and receptors.

AP2S1 and CSTB were negatively correlated with several chemokines and checkpoint-related genes (**Figures 6A–E**).

### 3.7 Functional enrichment of key genes

**Figure 7:**
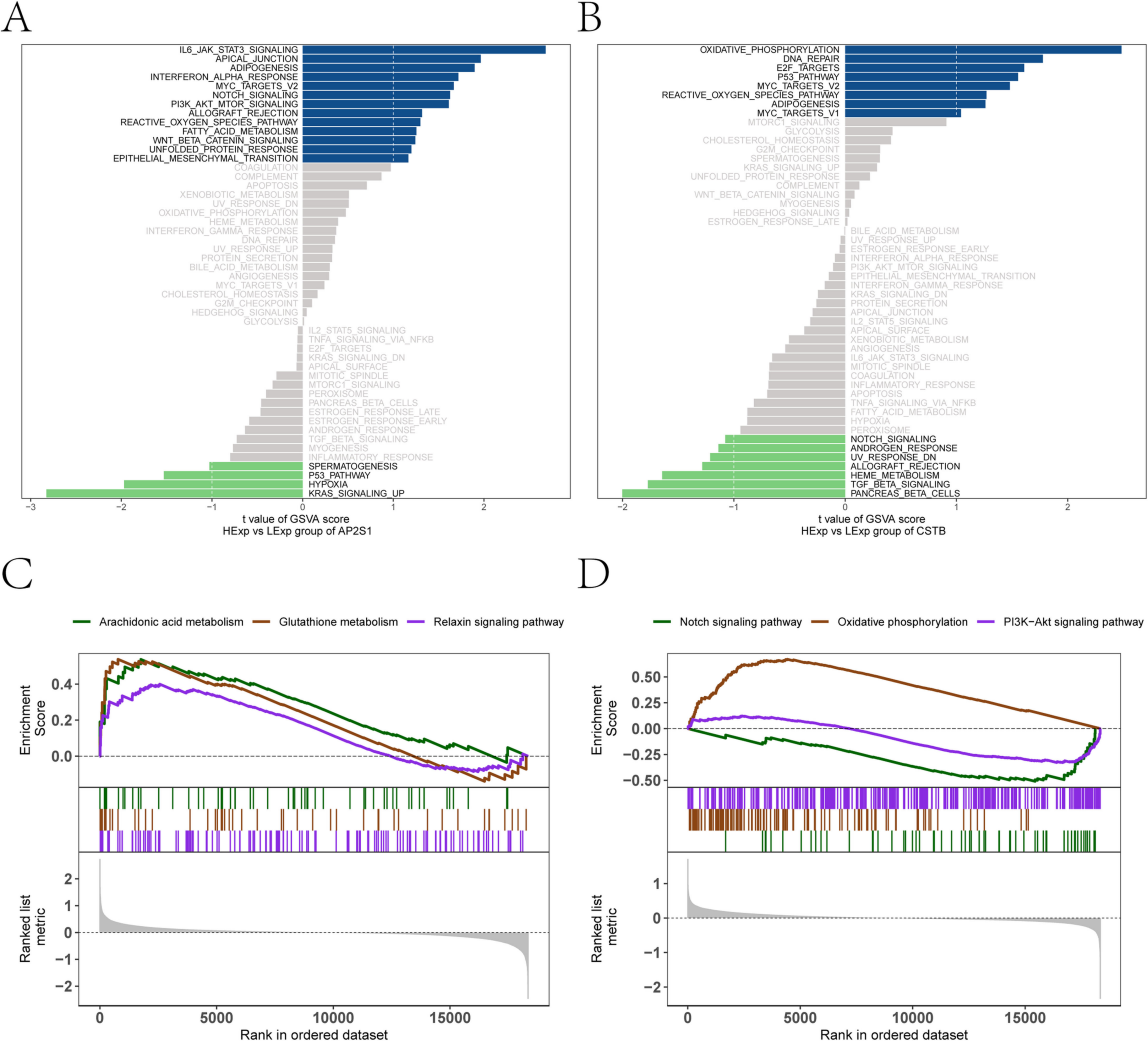
Functional enrichment analyses of key genes. **(A, B)** GSVA-based differential enrichment of MSigDB hallmark pathways between high- and low-expression groups for each feature gene (**(A)** AP2S1; **(B)** CSTB). Bars represent between-group differences in pathway activity. The x-axis shows the *t* statistic of GSVA scores, indicating the direction and magnitude of pathway shifts (positive values, higher activity in the high-expression group; negative values, higher activity in the low-expression group). Blue bars denote upregulated pathways, green bars denote downregulated pathways, and grey bars non-significant pathways. **(C, D)** GSEA highlighting pathways that differ significantly between high- and low-expression groups for each feature gene, with representative enriched KEGG pathways shown. The running curve indicates the enrichment score (ES), reflecting the distribution of pathway genes across the ranked gene list. Tick marks denote the positions of pathway genes within the ranked list; the grey density trace at the bottom shows the distribution of ranking metrics.

GSVA showed that AP2S1-high samples were associated with higher activity of IL6/JAK-STAT3 signaling, interferon response, and ROS pathways, whereas CSTB-high samples were associated with enrichment of oxidative phosphorylation and DNA repair (**Figures 7A, B**). GSEA showed consistent enrichment patterns, including arachidonic acid metabolism and glutathione metabolism in AP2S1-high samples and PI3K–Akt signaling in CSTB-high samples (**Figures 7C, D**).

### 3.8 Associations between key genes and established IDD-related regulators

**Figure 8:**
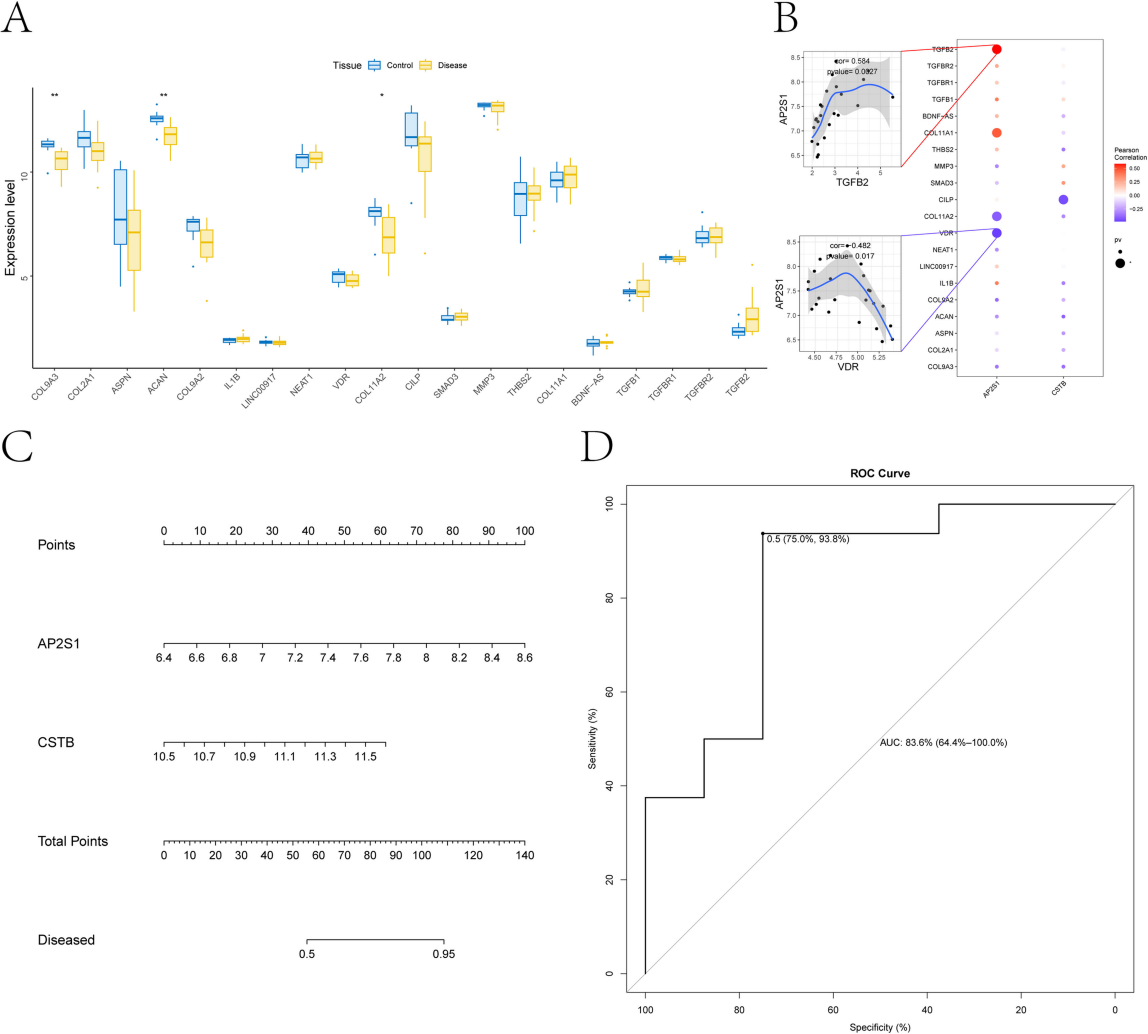
Disease relevance and nomogram-based prediction. **(A)** differential expression of established disease-regulatory genes between groups (blue, controls; yellow, disease). **(B)** correlation analysis between key genes and disease-associated genes; blue indicates negative correlations and red indicates positive correlations. **(C)** nomogram constructed from the selected feature genes to estimate individual disease probability; higher total points indicate greater predicted risk. **(D)** ROC curve evaluating the nomogram’s ability to discriminate disease status (AUC = 0.836). *p < 0.05; **p < 0.01.

To contextualize the prioritized genes within known IDD biology, we queried the GeneCards database to compile disease-associated genes linked to IDD. Candidates were ranked by relevance score, and the top 20 genes were retained for downstream analyses. Across patient-defined groups, we compared expression patterns of the key genes alongside this candidate set, and observed significant subtype-dependent differences for COL9A3, ACAN, and COL11A2. Correlation analyses further revealed expression associations between the key genes and established disease-related regulators. AP2S1 was positively correlated with TGFB2 (*r* = 0.584, *p* = 0.003) and inversely correlated with VDR (*r* = −0.482, *p* = 0.017) (**Figures 8A, B**).

### 3.9 Hub-gene model enables sample-level discrimination of IDD status

A logistic regression model based on the selected hub genes was visualized as a nomogram (**Figure 8C**). ROC analysis yielded an AUC of 0.836 in the discovery cohort (**Figure 8D**).

### 3.10 External validation of hub genes in an independent cohort

To assess whether the prioritized genes retained diagnostic signal outside the discovery setting, we analyzed an independent human NP cohort (GSE186542) comprising early degeneration (Pfirrmann grade I–III, n = 3) and late degeneration (grade IV–V, n = 3). ROC analysis showed supportive discriminatory performance for AP2S1 (AUC = 0.778, 95% CI: 0.291–1.000) and CSTB (AUC = 0.667, 95% CI: 0.013–1.000) (**Supplementary Figures 2A,B**). These results provide preliminary external support, although the wide confidence intervals reflect the limited cohort size.

### 3.11 Single-cell expression patterns and pseudotime dynamics of key genes

**Figure 9:**
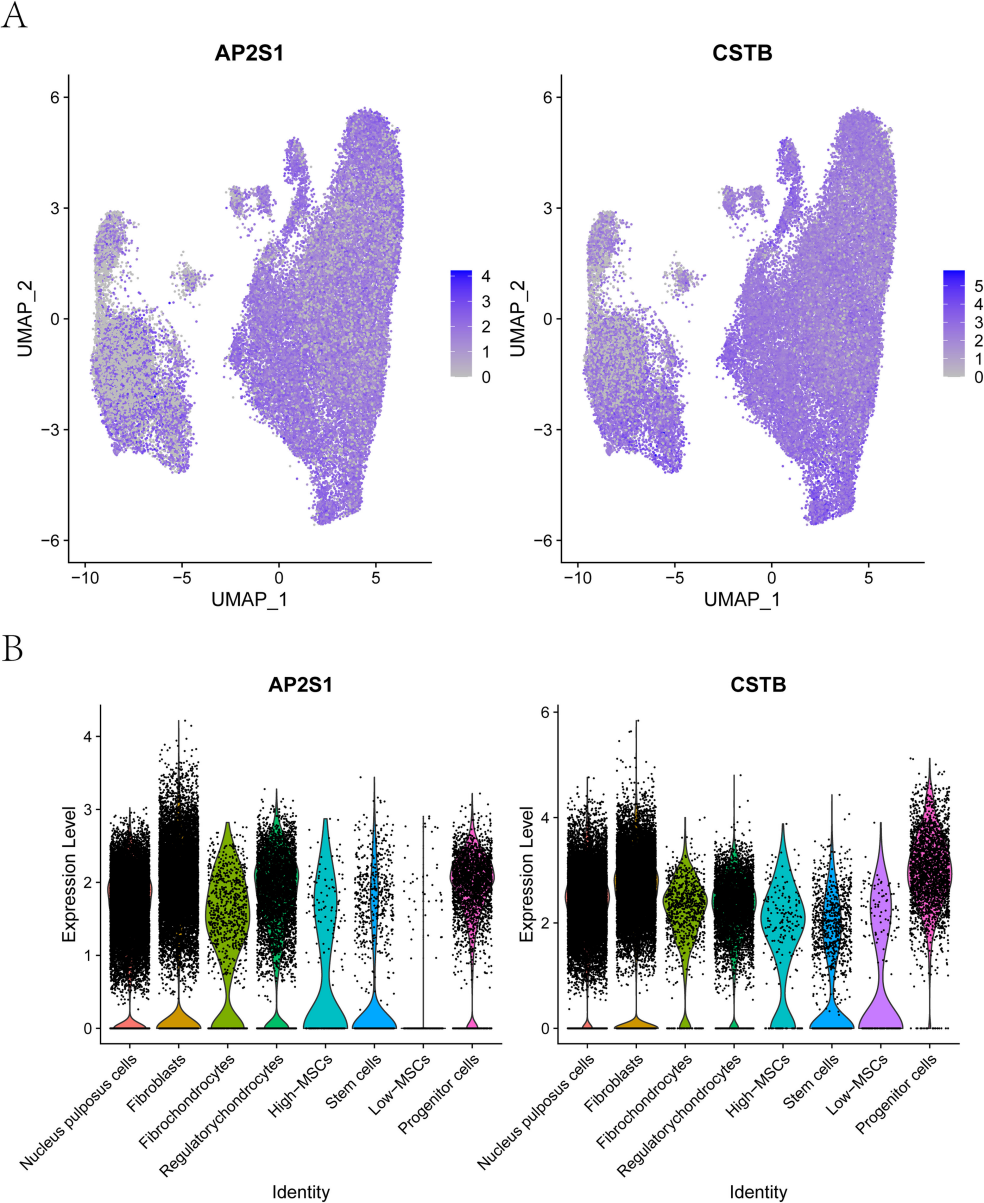
Single-cell expression patterns of key genes. **(A)** feature (scatter) plots showing the single-cell expression landscape of the key genes across the UMAP embedding. **(B)** violin plots summarizing the distribution of key-gene expression across cell populations at single-cell resolution.

We next examined the distribution of AP2S1 and CSTB across major cell populations, including MSCs, NP cells, fibrochondrocytes, fibroblasts, regulatory chondrocytes, stem cells, and progenitor cells. In addition to their differential expression between EV-score–high MSCs and EV-score–low MSCs, both genes were detectable in additional disc-resident cell types, suggesting that AP2S1 and CSTB signals were not restricted to MSCs and may reflect broader multicellular involvement (**Figures 9A, B**).

**Figure 10:**
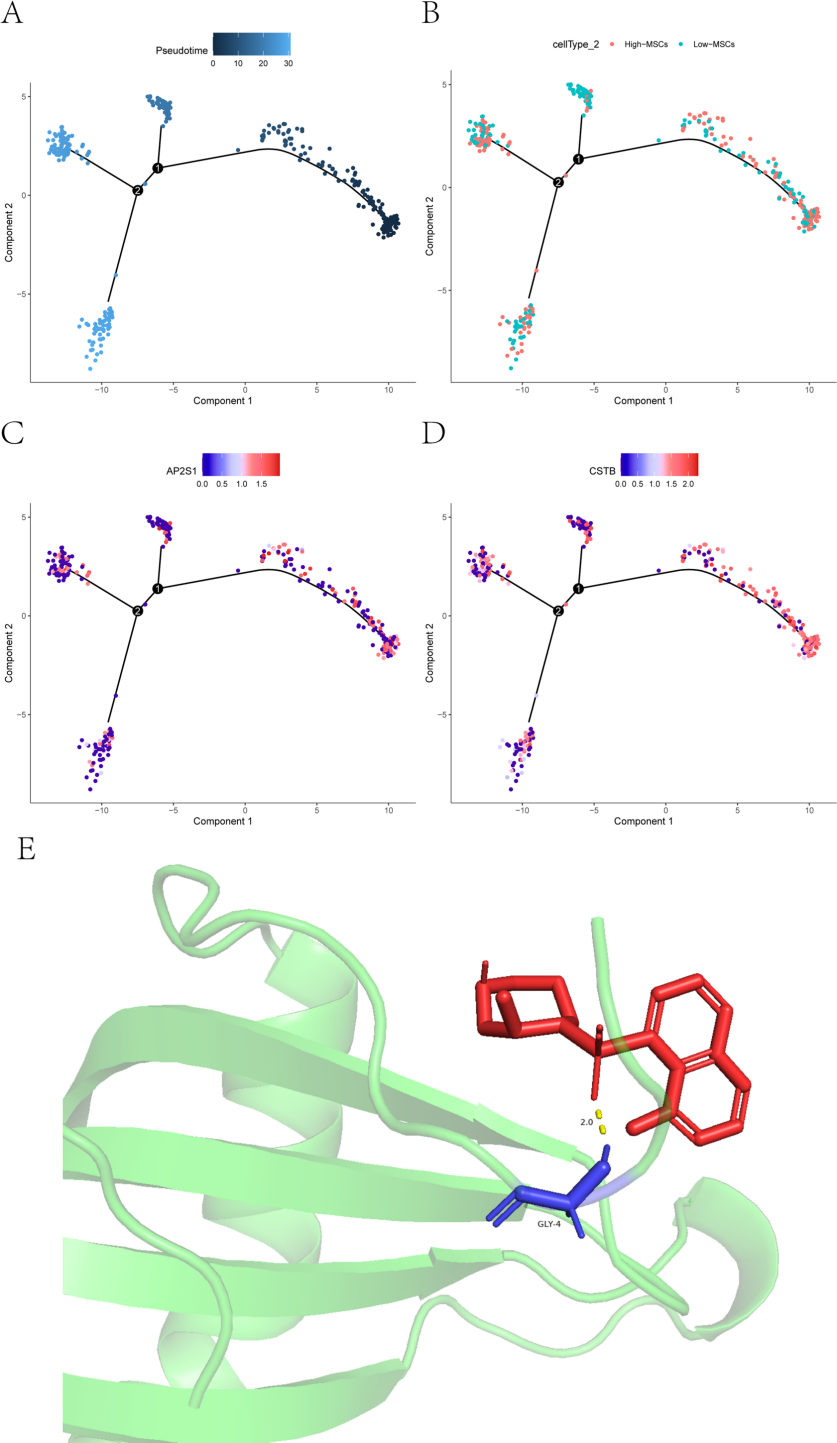
Pseudotime trajectory of MSC states and molecular docking. **(A)** distribution of cells along the inferred pseudotime trajectory. Color encodes pseudotime progression, with lighter shades indicating earlier states and darker shades indicating later states. **(B)** trajectory map annotated by cell state and subgroup identity (high-MSCs vs low-MSCs). **(C, D)** expression dynamics of feature genes along pseudotime. Color transitions from blue to red indicate increasing expression, highlighting potential roles of these genes during state transitions and fate-related processes. **(E)** representative molecular docking pose showing the three-dimensional interaction between a small-molecule ligand and the target protein. The protein backbone is shown in green, the ligand in red, and key interacting amino-acid residues in blue.

To resolve dynamic changes accompanying MSC state transitions, we performed pseudotime analysis comparing High-MSCs and Low-MSCs. MSCs aligned along an ordered trajectory, progressing from early to late states. Incorporating subgroup identity revealed that High-MSCs were enriched in the mid-to-late portion of the trajectory, whereas Low-MSCs predominated in earlier states, implying that these subpopulations occupy distinct positions along a maturation/activation continuum. This pattern points to developmental and functional heterogeneity within MSCs. EV-score–high MSCs may represent a more mature or activated state engaged in shaping disease-relevant microenvironmental processes. Consistently, AP2S1 and CSTB displayed clear, time-resolved expression dynamics along pseudotime, further supporting their functional relevance to MSC state regulation and IDD-associated biology (**Figures 10A–D**).

### 3.12 Functional divergence of MSCs stratified by key-gene expression

To quantify pathway activity at single-cell resolution, we applied AUCell to compute enrichment scores for multiple immune- and metabolism-related programs in each cell. MSCs were then stratified into high- and low-expression groups using the median expression of AP2S1 or CSTB, and pathway activities were compared systematically between groups. MSCs with high AP2S1 or CSTB expression exhibited increased activity of oxidative phosphorylation, MYC targets V1, and the ROS pathway, consistent with a more metabolically engaged, stress-responsive, and proliferative state. These results link key-gene expression to functional pathway activation and support the notion that AP2S1 and CSTB may jointly shape MSC states in the degenerative disc niche by modulating metabolic and stress-associated programs (**Supplementary Figure 2C**).

### 3.13 Molecular docking

We first screened AP2S1 and CSTB for putative druggable interactions. Only CSTB returned candidate small-molecule matches, and subsequent analyses therefore focused on evaluating CSTB–ligand binding potential. We selected the CSTB protein structure (UniProt ID: P04080) and the predicted compound ripasudil as the docking pair and performed molecular docking simulations to assess interaction stability and binding affinity. Docking indicated a binding free energy of −5.98 kcal/mol for CSTB (P04080) with ripasudil, consistent with a favorable interaction and suggesting a plausible binding interaction. These results provide preliminary computational support for CSTB as a pharmacologically tractable candidate in IDD (**Figure 10E**).

## 4 Discussion

In this study, we defined a single-cell MSC EV-program score and found that MSCs in IDD had higher scores than those in controls. By integrating LASSO with SVM–RFE feature selection, we prioritized five hub genes: AP2S1, CSTB, GSTP1, RPL28, and TSG101. AP2S1 and CSTB were upregulated in degenerative samples and were supported by preliminary RT–qPCR validation in an *in vitro* inflammatory NP-cell model. CellChat-based communication mapping and pseudotime inference consistently positioned high-score MSCs in a state of heightened signal reception, while GSVA/GSEA linked the associated transcriptional program to key immunometabolic and stress-response axes, including IL6–JAK–STAT3, PI3K–Akt, p53 signaling, oxidative phosphorylation, and ROS stress. A nomogram derived from the signature showed within-cohort discriminatory performance (AUC = 0.836), underscoring the diagnostic potential of this gene combination for distinguishing degenerative from non-degenerative states. Molecular docking suggested that the small molecule ripasudil can engage CSTB (predicted binding energy ≈ −5.98 kcal/mol), providing a plausible pharmacological hypothesis for follow-up testing. Our data delineate an IDD-associated MSC EV-linked regulatory program; therapeutic causality of exogenous MSC-EVs remains to be tested in dedicated functional systems.

Prior work provides context for these findings. AP2S1 encodes the σ subunit of the AP-2 adaptor complex, a core component of receptor-mediated endocytosis, particularly clathrin-mediated endocytosis (CME). Multiple studies have shown that a wide range of cells—including neuronal and cancer cells—internalize EV cargos or EVs predominantly through CME-dependent routes (17). The upregulation of AP2S1 in MSCs with high EV-program scores therefore suggests that these cells may have an enhanced capacity to take up MSC-EVs and other extracellular cues (e.g., growth-factor–linked signals), enabling heightened responsiveness within the degenerative niche. In line with this notion, tumor-derived MSC-EVs have been reported to enter MSCs via AP-2–associated pathways and subsequently reshape MSC transcriptional programs (17). In parallel, TSG101, a key component of the ESCRT machinery, is widely used as a canonical EV marker (11). Beyond TSG101, other commonly used EV-associated markers include the tetraspanin panel (CD63, CD81, and CD9) and syntenin-1 (SDCBP). These markers are frequently used in combination because they capture complementary aspects of EV biology: tetraspanins are membrane-associated EV markers, whereas TSG101 and syntenin-1 are linked to endosomal/ESCRT-related EV biogenesis. Following MISEV2018 recommendations, EV characterization is generally strengthened by evaluating multiple marker categories rather than relying on a single marker. In this context, our emphasis on TSG101 should be interpreted within a broader EV-marker framework that includes CD63/CD81/CD9 and SDCBP. Its elevated expression in high-score MSCs is consistent with a more active vesicle biogenesis/secretion state and aligns with our CellChat results positioning these cells as prominent signal receivers. Coordinated shifts in AP2S1 and TSG101 point to an EV-score–high MSC subset that is more engaged at both ends of vesicle trafficking—endocytic uptake and exosomal output—and may therefore occupy a pivotal role in intercellular communication during disc degeneration.

CSTB (cystatin B; stefin B) was elevated in high-score MSCs and may provide a mechanistic entry point into immunometabolic regulation. CSTB is known to protect cells by restraining cysteine cathepsin activity, and CSTB deficiency has been linked to excessive NLRP3 inflammasome activation alongside increased ROS accumulation (18, 19). In the context of IDD, NLRP3-driven pyroptosis is increasingly recognized as a contributor to tissue inflammation and cellular injury (19). Our enrichment analyses connected CSTB expression to oxidative phosphorylation and ROS-related stress pathways, raising the possibility that CSTB may contribute to redox defense in EV-score–high MSCs, thereby buffering oxidative stress in the NP niche and tempering downstream inflammatory cascades. GSTP1, a glutathione S-transferase family member, showed a related pattern. Its upregulation is consistent with enhanced capacity for ROS detoxification, which may help preserve the vesicle biogenesis and secretion programs required for MSC-EV-mediated paracrine activity. These observations fit the broader immunometabolic landscape of IDD, where oxidative stress induced by immune cells and inflammatory mediators (e.g., IL-1β and TNF-α) amplifies NP-cell damage (19), whereas antioxidant circuits can counter this trajectory. CSTB and GSTP1 may participate in MSC-EV-linked regulation of the IL-1β/NLRP3 axis and in the containment of ROS burden, supporting their prioritization as candidate targets for stabilizing the degenerative disc microenvironment.

RPL28 encodes ribosomal protein L28. Its higher expression may reflect augmented translational capacity and secretory throughput in high-score MSCs—consistent with the notion that an MSC-EV-active state requires increased production of vesicle-associated and cargo-related proteins. Although ribosomal proteins have been less studied in IDD, our data raise the possibility that specific ribosomal components may help sustain heightened biosynthetic demand under stress conditions. We also observed enrichment of IL-6–JAK-STAT3 and PI3K–Akt signaling in high-score MSCs (see enrichment outputs and figure annotations). This aligns with prior evidence that IL-6/JAK-STAT3 signaling in IDD promotes inflammatory mediator expression and shapes immune-cell activity (8, 19). The PI3K–Akt pathway, in turn, is closely tied to cell survival and metabolic rewiring and may be engaged in MSCs participating in tissue repair programs (6, 19). Consistent with these pathway-level signals, our immune-infiltration analyses linked the key genes to a broad set of chemokines (including CCL and CXCL family members) and immune-checkpoint molecules (e.g., PD-L1 and CTLA-4), suggesting that MSC-EV programs may influence immune-cell recruitment and activation in the degenerative niche through cytokine and checkpoint modulation. These results are consistent with immunometabolic studies showing that immune-cell function is linked to metabolic state (6), and MSCs and their MSC-EVs may act as local immunoregulatory agents in part by reshaping metabolic circuitry.

This analysis extends prior IDD and MSC-EV studies by linking an EV-program score to immune and metabolic networks. A substantial body of evidence supports regenerative and anti-inflammatory effects of MSC-derived EVs in IDD. For example, the review by Bhujel and colleagues summarizes that MSC-EVs can promote NP cell proliferation, restore matrix anabolism, and suppress apoptosis (12). Zhao and colleagues further showed that MSC-EVs released by MSCs after hypoxic and inflammatory priming deliver miR-221-3p to inhibit the DDIT4/NF-κB axis, thereby attenuating NP-cell senescence (14). Our findings are consistent with these reports in supporting the protective potential of MSC-EV programs; we extend prior work by quantifying an EV-associated program at single-cell resolution and by linking this functional state to downstream targets embedded within immunometabolic networks—an analytical layer largely absent from prior studies. In parallel, single-cell studies have increasingly highlighted the heterogeneity of disc-resident cells. Ling and colleagues delineated multiple NP cell states and inflammatory programs and reported MIF/NF-κB-mediated signaling between macrophages and NP progenitors (9). Zhang and colleagues further described context-dependent roles of local immune populations, including SPP1+ macrophages, during degeneration (9, 15). Our CellChat analyses complement this emerging communication atlas by showing that EV-score–high MSCs are positioned primarily as signal recipients within the network, consistent with a model in which they sense inflammatory or trophic cues from immune cells and, in turn, remodel the niche through MSC-EV-mediated feedback. This interpretation aligns with the prevailing view that the immune milieu surrounding the intervertebral disc can exert both injurious and reparative influences, depending on context and balance (8, 9). By integrating single-cell profiling with machine-learning prioritization, we not only identify genes tightly linked to MSC-EV programs but also outline a plausible mechanism whereby these regulators operate along an “immune–inflammatory–metabolic communication” axis to shape IDD trajectories. This approach provides complementary insight to prior multi-omics and network-based efforts aimed at nominating diagnostic factors and therapeutic targets for IDD (10, 20). A direct translational next step is to link the EV-score program to quantitative EV phenotypes (particle number, size distribution, EV markers, cargo) and to recipient-cell functional outputs in IDD-relevant assays. Such experiments, designed according to MISEV2018 recommendations (EV isolation/characterization, dose reporting, functional attribution controls), would enable causal testing of whether manipulating AP2S1/CSTB alters MSC-EV biogenesis/uptake and downstream disc-cell immunometabolic states (21).

### 4.1 Limitations and future directions

This study has several limitations. The primary single-cell analysis was based on GSE230809, which included 8 IDD samples and 3 controls; this sample size may limit the generalizability of the MSC EV-program score and hub-gene prioritization. The EV-program score is transcriptome-inferred and was not matched to direct EV measurements, such as particle number, size distribution, EV-marker profiling, cargo analysis, or proteomic validation. The relationship between the score and actual EV abundance, secretion rate, or biological potency therefore remains unresolved. Most conclusions are based on transcript-level associations. Although AP2S1 and CSTB were supported by RT–qPCR in an inflammatory NP-cell model, their protein-level changes and causal roles in MSC-EV biology or IDD progression still require gain- and loss-of-function testing. CellChat, GSVA/GSEA, immune-infiltration analysis, and molecular docking provide computational or correlative evidence rather than mechanistic proof. The *in vitro* NP-cell model does not reproduce the multicellular, immune, biomechanical, and nutritional features of the native degenerative disc niche. Sex, age, degeneration grade, and clinical heterogeneity could not be stratified because the public datasets lacked complete annotations. The external validation in GSE186542 provides preliminary support because of its small sample size. Future work should test the EV-program score in larger multicenter and spatially resolved cohorts, quantify EV phenotypes directly, perturb AP2S1 and CSTB in MSCs, and evaluate downstream effects on EV production, cargo composition, NP-cell inflammation, redox stress, and disc degeneration in cell and animal models. Independent clinical cohorts should also be used to examine AP2S1/CSTB-associated signals in blood or disc-fluid specimens and to compare the current classifier with existing clinical or biomarker-based models.

## 5 Conclusions

In intervertebral disc degeneration (IDD), mesenchymal stromal cells (MSCs) shift toward an EV-program–high state that is tightly coupled to immune communication and immunometabolic stress. Using a transcriptome-inferred MSC EV–associated program score, we observed increased EV-program activity in IDD MSCs and found that EV-score–high MSCs exhibit enhanced incoming signaling and interaction potential in CellChat networks, consistent with an immune cue–responsive phenotype.

By integrating LASSO with SVM-RFE, we prioritized five hub genes (AP2S1, CSTB, GSTP1, RPL28, and TSG101). AP2S1 and CSTB were supported by RT–qPCR under inflammatory stimulation and displayed dynamic patterns along MSC pseudotime trajectories. Pathway analyses linked the hub-gene program to immune–metabolic axes, including IL6/JAK–STAT3 and interferon-related signaling, together with oxidative phosphorylation and reactive oxygen species (ROS) stress, and showed coordinated associations with immune mediators such as chemokines and checkpoint-related genes. A hub-gene model showed within-cohort discrimination of IDD status (AUC = 0.836), and docking suggested a plausible interaction between CSTB and ripasudil (−5.98 kcal/mol), providing a testable pharmacologic hypothesis.

These results place MSC EV–associated transcriptional programs within a disc immune microenvironment framework and nominate AP2S1 and CSTB as tractable nodes linking EV-program states to immune signaling and metabolic stress in IDD.

## Data Availability

The datasets analyzed in this study are publicly available from GEO under accession numbers GSE230809, GSE70362, and GSE186542.
