# Correction: Single-cell extracellular vesicle-program scoring maps immunometabolic rewiring and immune crosstalk of mesenchymal stromal cells in intervertebral disc degeneration, prioritizing AP2S1 and CSTB

**DOI:** 10.3389/fimmu.2026.1893698

**Published:** 2026-06-05

**Authors:** Fengyu Ma, Kelv Shen, Ziqiang Wu, Hanshi Yang, Mingzhe Yu, Zhendong Huang, Fengxuan Han, Zhengfeng Lu

**Affiliations:** 1 Department of Orthopedics, The Second Affiliated Hospital of Soochow University, Suzhou, Jiangsu, China; 2 Department of Spinal Surgery, People’s Hospital of Rizhao, Rizhao, Shandong, China; 3 Medical 3D Printing Center, Orthopedic Institute, Department of Orthopedic Surgery, The First Affiliated Hospital, School of Basic Medical Sciences, Interdisciplinary Innovation Center for Nanomedicine, MOE Key Laboratory of Geriatric Diseases and Immunology, Suzhou Medical College, Soochow University, Suzhou, Jiangsu, China; 4 Children’s Hospital of Soochow University, Suzhou, Jiangsu, China

**Keywords:** immunometabolic reprogramming, intervertebral disc degeneration, machine learning, mesenchymal stromal cells, MSC EV-program scoring, single-cell RNA sequencing

In the published article, two textual errors were identified in the **Results** section. First, an in-text citation to **Supplementary Figure 1** was omitted in Section 3.1, “*Single-cell atlas of NP tissues in IDD*.” Second, a citation to **Figure 2C** was incorrectly included in Section 3.2, “*EV-associated program scoring identifies an MSC-shift in IDD*,” after the sentence reporting the 16 EV-score–linked genes. **Figure 2C** shows scoring distributions rather than the 16 EV-score–linked genes.

A correction has been made to Section 3.1, “*Single-cell atlas of NP tissues in IDD*,” paragraph 1. The original sentence was:

“After quality control (≥200 genes per cell; mitochondrial fraction cutoff as specified in Methods), cells were retained for downstream analyses.”

The corrected sentence reads:

“After quality control (≥200 genes per cell; mitochondrial fraction cutoff as specified in Methods), cells were retained for downstream analyses (**Supplementary Figure 1**).”

A correction has also been made to Section 3.2, “*EV-associated program scoring identifies an MSC-shift in IDD*,” paragraph 1. The original sentence was:

“Using |avg_log2FC| > 0.500 and FDR < 0.050, we obtained 16 EV-score–linked genes (**Figure 2C**).”

The corrected sentence reads:

“Using |avg_log2FC| > 0.500 and FDR < 0.050, we obtained 16 EV-score–linked genes.”

The authors apologize for these errors and state that these corrections do not change the data, analyses, results, interpretation, or conclusions of the article.

The original version of this article has been updated.

