# Supplementary material for: Single-cell extracellular vesicle-program scoring maps immunometabolic rewiring and immune crosstalk of mesenchymal stromal cells in intervertebral disc degeneration, prioritizing AP2S1 and CSTB
Source: Front Immunol. 2026 May 22;17:1820174. doi: 10.3389/fimmu.2026.1820174 (PMC13236619; doi:10.3389/fimmu.2026.1820174)
Supplement: Supplementary file 1 [file DataSheet1.docx]

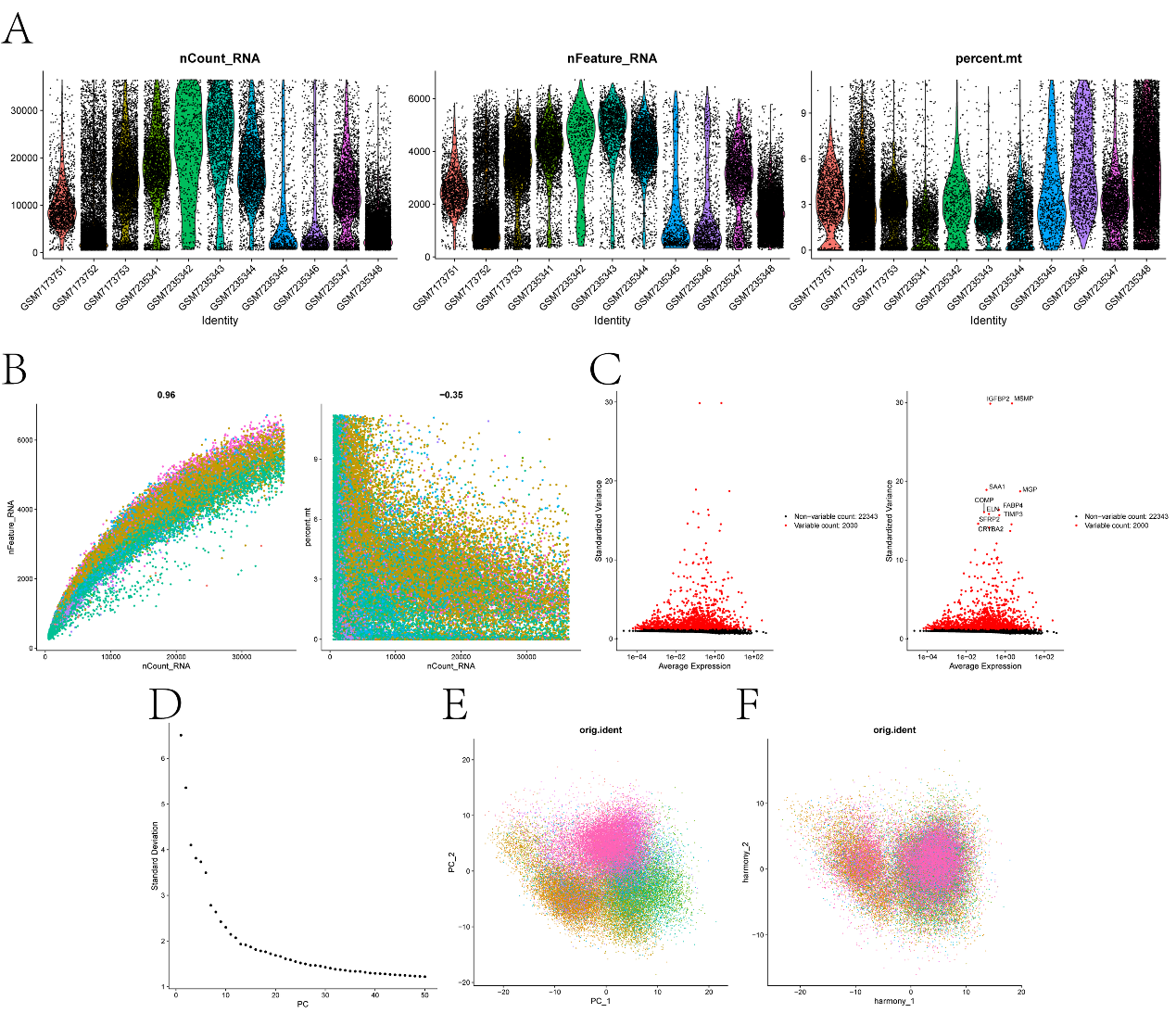


**Supplementary Fig. 1. Single-cell preprocessing and quality control. (A)** Summary of retained cells, detected genes, and sequencing depth across samples. **(B)** Relationships among sequencing depth, mitochondrial transcript fraction, and gene complexity. Each point represents one cell. **(C)** Highly variable genes across cells. **(D)** Scree plot showing variance explained by each principal component (PC). **(E–F)** PCA embeddings colored by sample identity.


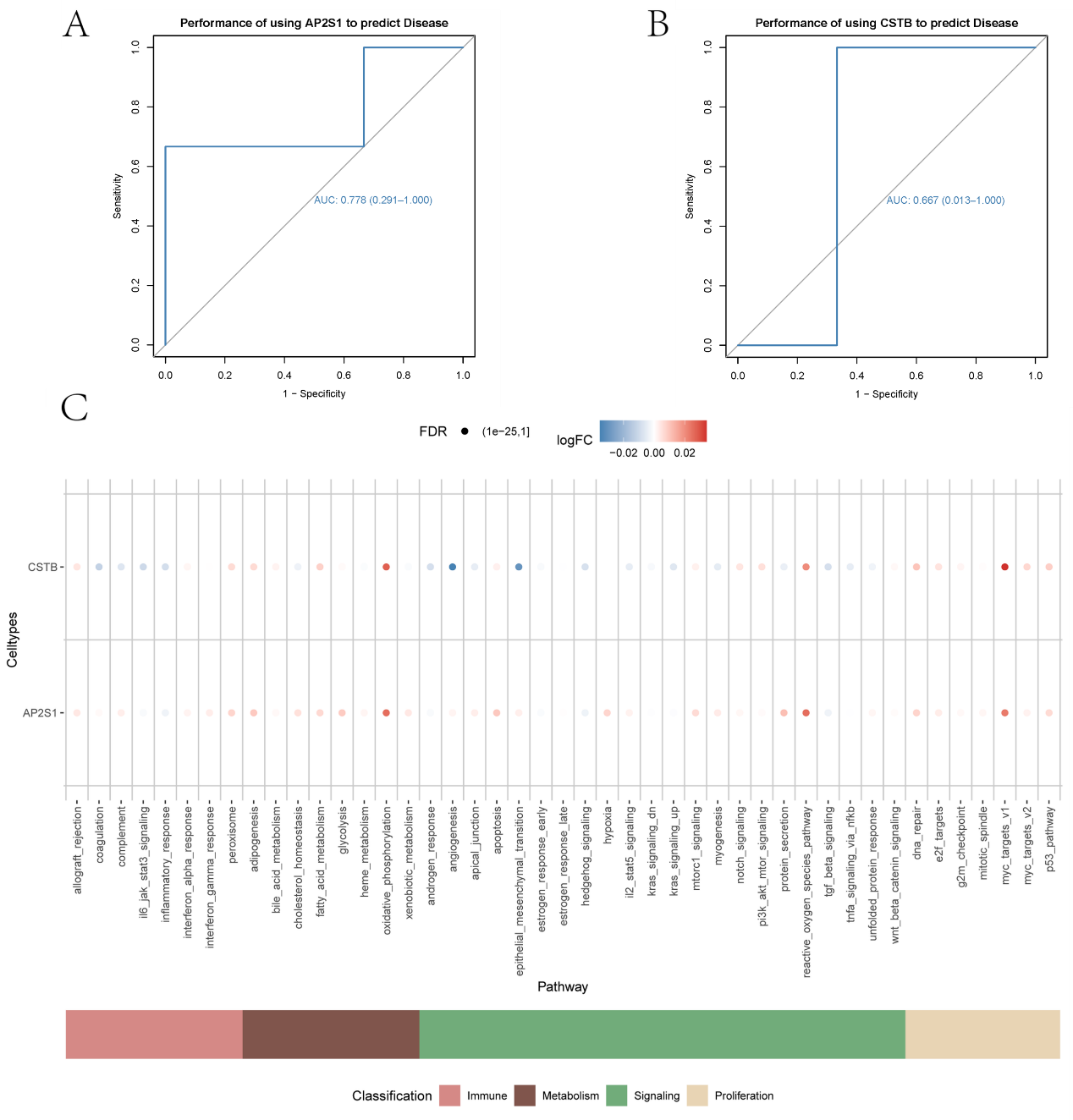


**Supplementary Fig. 2. External validation of AP2S1 and CSTB and pathway changes associated with these genes. (A–B)** ROC curves for AP2S1 and CSTB in GSE186542, comparing early degeneration (Pfirrmann grade I–III, n = 3) with late degeneration (grade IV–V, n = 3). AUC values were 0.778 and 0.667, respectively. **(C)** Dot plot of pathway-level changes associated with AP2S1 and CSTB across selected Hallmark pathways. Dot color indicates logFC (blue, lower; red, higher), and dot size indicates FDR.
